# Patterns of tsetse abundance and trypanosome infection rates among habitats of surveyed villages in Maasai steppe of northern Tanzania

**DOI:** 10.1186/s40249-017-0340-0

**Published:** 2017-09-04

**Authors:** Anibariki Ngonyoka, Paul S. Gwakisa, Anna B. Estes, Linda P. Salekwa, Happiness J. Nnko, Peter J. Hudson, Isabella M. Cattadori

**Affiliations:** 1 School of Life Sciences and Bioengineering, Nelson Mandela African Institution of Science and Technology, P.O. Box 447, Arusha, Tanzania; 2 Genome Sciences Center, Department of Microbiology, Parasitology and Immunology. College of Veterinary and Medical Sciences, Sokoine University of Agriculture, Morogoro, Tanzania; 3 Centre for Infectious Disease Dynamics, The Huck Institutes of the Life Sciences, The Pennsylvania State University, University Park, USA; 4 Department of Conservation Biology, School of Biological Sciences, University of Dodoma, Dodoma, Tanzania; 5 Department of Geography and Environmental studies, University of Dodoma, Dodoma, Tanzania

**Keywords:** Habitat variability, Tsetse fly, Host availability, Infection rate, Trypanosomes

## Abstract

**Background:**

Changes of land cover modify the characteristics of habitat, host-vector interaction and consequently infection rates of disease causing agents. In this paper, we report variations in tsetse distribution patterns, abundance and infection rates in relation to habitat types and age in the Maasai Steppe of northern Tanzania. In Africa, Tsetse-transmitted trypanosomiasis negatively impacted human life where about 40 million people are at risk of contracting the disease with dramatic socio-economical consequences, for instance, loss of livestock, animal productivity, and manpower.

**Methods:**

We trapped tsetse flies in dry and wet seasons between October 2014 and May 2015 in selected habitats across four villages: Emboreet, Loiborsireet, Kimotorok and Oltukai adjacent to protected areas. Data collected include number and species of tsetse flies caught in baited traps, PCR identification of trypanosome species and extraction of monitored Normalized Difference Vegetation Index (NDVI) data from Moderate Resolution Imaging Spectrometer (MODIS).

**Results:**

Our findings demonstrate the variation of tsetse fly species abundance and infection rates among habitats in surveyed villages in relation to NDVI and host abundance. Results have shown higher tsetse fly abundance in Acacia-swampy ecotone and riverine habitats for Emboreet and other villages, respectively. Tsetse abundance was inconsistent among habitats in different villages. Emboreet was highly infested with *Glossina swynnertoni* (68%) in ecotone and swampy habitats followed by *G. morsitans* (28%) and *G. pallidipes* (4%) in riverine habitat. In the remaining villages, the dominant tsetse fly species by 95% was *G. pallidipes* in all habitats. *Trypanosoma vivax* was the most prevalent species in all infected flies (95%) with few observations of co-infections (with *T. congolense* or *T. brucei*).

**Conclusions:**

The findings of this study provide a framework to mapping hotspots of tsetse infestation and trypanosomiasis infection and enhance the communities to plan for effective control of trypanosomiasis.

**Electronic supplementary material:**

The online version of this article (doi:10.1186/s40249-017-0340-0) contains supplementary material, which is available to authorized users.

## Multilingual abstracts

Please see Additional file 1 for translation of the abstract into the six official working languages of the United Nations.

## Background

The epidemiology of trypanosomiasis is driven by the transmission of multiple species of protozoa of the genus *Trypanosoma* by various species of vectors, human and livestock hosts, and a large number of wild animal species that act as the major reservoir hosts [1, 2]. In Africa, tsetse-transmitted trypanosomiasis negatively impacted human life where about 40 million people are at risk of contracting the disease with dramatic socio-economical consequences, for instance, loss of livestock, animal productivity and manpower [3, 4].

The important pre-requisite towards the understanding of the transmission dynamics of the disease is the quantification of trypanosome infection rate and abundance in the vectors and susceptible hosts [5, 6]. Multiple trypanosome species are found to infect animals with varying degrees of adaptation among host species [7, 8]. Geospatial data in sub-Saharan Africa shows that it is extensively infested by various species of tsetse and hence potentially transmit the disease to both humans and animals [9]. Literature shows distinct patterns of spatial prevalence in humans caused by *Trypanosoma brucei rhodensiense* and *T. brucei gambiense* for East and West Africa [10]. *T. b. rhodesiense* is known to cause acute and rapidly progressive disease, and widely affecting livestock and wildlife but occasionally humans [11]. Gambian trypanosomiasis is a chronic infection which accounts for 98% human reported cases, whereas, *T. b. rhodesiense* has the potential for epidemic outbreaks in humans [12, 13].

The distribution and abundance of vectors and the availability of hosts invariably influence the prevalence of *Trypanosoma* species among habitats [14, 15]. These components play a critical role in the identification of the “*pathogenic landscapes*”, which takes into account the pathogenicity of the parasite, the susceptibility of the host, both livestock and human, and how they vary over time to affect disease persistence [15].

Tsetse flies are haematophagous vectors that depend on host species availability to feed pregnant female as well as the adult tsetse [16]. This provides a chance for circulation of trypanosomes between wild animals, human, livestock, and tsetse flies and hence transmission of the disease [17]. Habitat plays a key role in supporting the parasite through the provision of microclimatic conditions important during the incubation period, before becoming infective [18]. Habitat is also important for vector breeding, resting and refuging during adverse climatic conditions as it provides suitable microclimatic settings for pupal development and mature fly survival [19].

The Maasai steppe is reported to be infested by tsetse flies and trypanosomiasis since colonial time [20]. Maasai’s livestock often coexist with wild animals through sharing grazing areas. In recent years the Maasai people have integrated crop cultivation as a means of their livelihood and become sedentary [21]. This has restricted the areas for grazing and increased interaction with wildlife, especially towards the edge of protected areas, with the potential for an increase of susceptible cases, and infection risk. Given the increased sharing of the habitat for grazing, it is important to determine the spatial and temporal dynamics of both vector and parasite in relation to habitat types in order to identify the infected landscape at higher risk of disease transmission and plan for both control and grazing which aims to reduce prevalence as well as transmission.

The scope of this paper was to examine the spatial abundance and seasonal changes in tsetse flies and trypanosomes prevalence in relation to the habitats present in the Maasai steppe of Northern Tanzania. Specifically, three questions were considered: How tsetse fly abundance varies with seasons across habitats of the surveyed villages? Do infection rates of various species of trypanosomes vary with habitats across seasons in the surveyed villages? What is the average age of vector infection by species and habitat?

## Methods

### Study area

Maasai steppe (3°40′ and 4°35′ South, 35°50′ and 36°20′E) includes protected areas; Tarangire National Park (TNP), Manyara National Park (MNP) and Simanjiro plains with semi arid vast open wooded savannah and seasonal swampy areas in northern part of Tanzania. The area is inhabited by livestock keepers dominated by Maasai people. The livelihood of the Maasai is centered around livestock management while crop cultivation is used as means to supplement the basic needs. The study area is characterized by bimodal rainfall pattern with short rain between October and December and long rain between March and April [22]. The Maasai are known to co-exist with abundant wild animals which exhibit seasonal migrations to and from the community grazing area [23]. The steppe is documented to be regularly infested by various species of tsetse and trypanosomiasis cases from the colonial time [20] until recent years [14, 24].

In this study, we sampled four main habitats across the villages close to TNP, MNP and Manyara ranch. The habitats include open-woodland swampy ecotone habitat, Swampy habitats (The wooded grassland area which retains waters above the surface during the rainy season and remains moist with green grass throughout the dry season), Open woodland (scattered trees with grassland undercover) and riverine habitat.

### Host counts

Data on host availability for both wild animals and livestock were collected by recording the species and number of animals in each habitat detected up to a square plot of 100 × 100 m around the geo-referenced fly traps every sampling day. The mean number of each host species was calculated and hence compared with its corresponding tsetse relative abundance (proportions) in each trap in a habitat. The relative abundance of the collected host species were compared with corresponding tsetse catches from traps.

### Acquisition of land cover satellite data

The extracted normalized difference vegetation index (NDVI) values from Moderate Resolution Imaging Spectrometer (MODIS) from the Terra and Aqua satellites, The Earth Resources Observation and Science Center (EROS) of the United States Geological Survey (https://glovis.usgs.gov/) for different sites that match with the time of tsetse sampling was used for the analysis. NDVI are derived from the visible red wavelength (620–670 nm) and near infrared wavelength (841–876 nm) of the electromagnetic spectrum [25, 26]. The spatial and temporal resolution of downloaded images was 250 m and in a composite of a 16 day period.

### Entomological survey

**Fig. 1:**
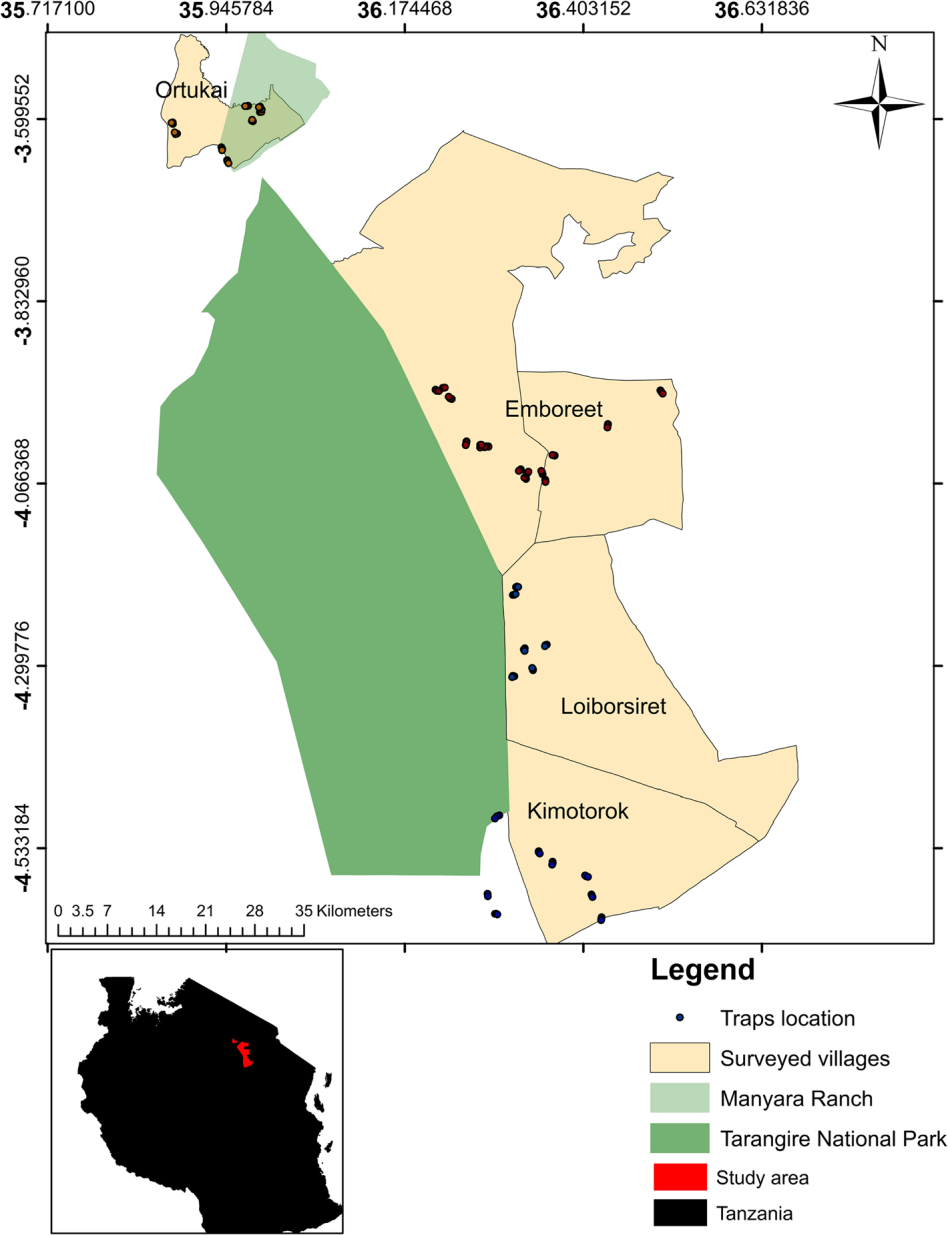
Locations of traps and villages during entomological survey in Maasai Steppe, Northern Tanzania

The entomological survey was conducted by sampling tsetse flies in different habitats between July 2014 and November 2015. Four villages along the edge of protected areas; the Tarangire National Park and Manyara Ranch boundary were selected for sampling. Simple random selection was used to choose the four villages from other villages surrounding the protected areas. These were namely, Emboreet, Loiborsireet, Kimotorok, and Oltukai (Fig. 1). The minimum number of traps used per village was 24 and at least three traps were set per habitat. Traps were baited with 4-methyl phenol (1 g/h) 3-n-propylphenol (0.1 mg/h) 1-octen-3-ol (0.5 mg/h) and acetone (100 mg/h) [27] were placed in the same geo-referenced points in each habitat for six days of one month during the dry and wet season. Tsetse flies were collected daily from the geo-referenced traps and were identified according to their species, sex, and age [28] and preserved in collection tubes with pure ethanol for the molecular identification of trypanosome species.

### DNA extraction and identification of trypanosomes

**Fig. 2:**
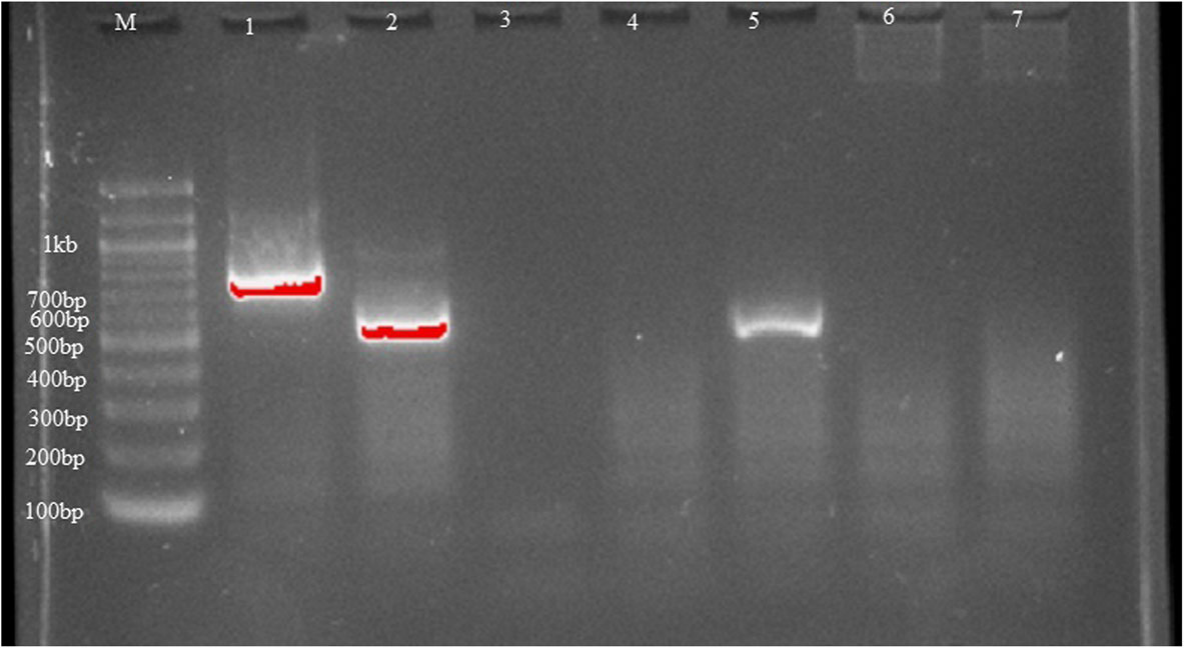
Trypanosome DNA product by ITS - PCR from Maasai steppe tsetse samples**.** Number 1 is *T. congolense* ~ 700 and number 5 is a *Typanozoon* members (*T. brucei*) ~ 480 bp. Number No. 2 is positive control for *T. brucei* and number 3 is negative control. M = 1 kb DNA ladder

The collected samples of individual flies were dried and then crushed in the laboratory using hand pestle. The DNA extraction procedures followed the Ammonium Acetate Precipitation protocol [29, 30]. The DNA samples were stored at −20 °C for further analysis. DNA extraction was followed by convectional PCR for identification of trypanosome species circulating in tsetse flies collected in the study area. Primers targeting the Internal Transcribed Spacer 1 (ITS1) gene of trypanosomes were used for screening the tsetse flies DNA. Tsetse DNA was analyzed in pools constituted of 10 DNA samples each and later individual samples of positive pools. The reaction was performed in a total volume of 15 μl containing 7. 5 μl Dream Taq master mix, 200 nM of forward and reverse primers and 3.9 μl of nuclease free water. The ITS 1 primer sequences used were CF 5′-CCG GAA GTT CAC CGA TAT TG-3′ and BR 5′-TTG CTG CGT TCT TCA ACG AA-3’ [31]. The cycling conditions were: initial denaturation at 94 °C for 3 min followed by 30 cycles of denaturation at 94 °C for 30 s, annealing at 55 °C for 30 s, extension at 70 °C for 30 s and lastly final extension at 72 °C for 10 min. The PCR products were separated on 2% GR- green stained agarose gels and positive results were identified based on the size of the PCR amplicons. The amplicon sizes differ between species of trypanosomes whereby, *Trypanosoma brucei gives* 480 bp and *Trypanosoma congolense savannah* 700 bp (Fig. 2) while *Trypanosoma vivax* 250 bp.

### Data analysis

The data were analyzed using the R statistical software [32] for analytical and descriptive statistics. In this work, we showed variation of three tsetse species among habitats and season for each village, presented using lattice and grid extra of ggplot2 of R libraries. The same libraries have been used to present the relationship between the infection rates and tsetse species and among habitats. Linear Mixed Effect Models (LME, fit by maximum likelihood) were used to examine the relationship between tsetse species abundance, as a response, and habitat and season, included as independent variables. The abundance of tsetse species is considered as log transformed mean number of tsetse caught per trap per day in a habitat.

Sampling site or habitat, when necessary, was included as a random factor to account for variability among sites and the sampling of the same site every month. In this paper, we used linear mixed models because it models associated variables while retaining a normal distribution of the errors [33]. In addition, the random variables are added to the linear predictor as the extension of linear models.

However, infection among seasons was analysed using the R software. The mean age of fly survival and the mean age at which the fly become infective were calculated using the wingfrey technique described in the training manual for tsetse control personnel [28]. Kimotorok village was excluded from the analysis because of very low fly abundances.

## Results

### Abundance of tsetse among habitats and across villages during extensive survey

**Fig. 3:**
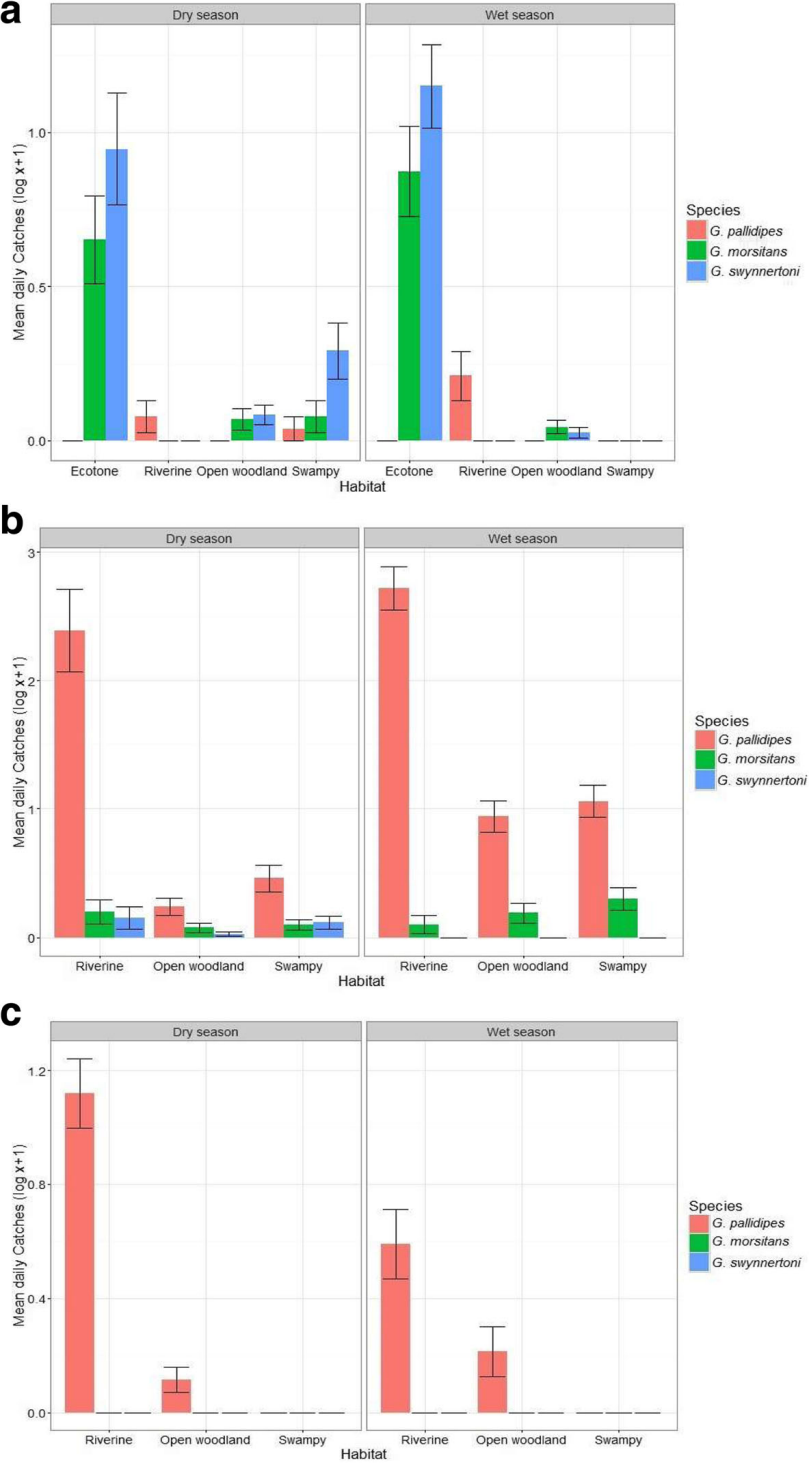
Geometric mean daily catches of tsetse species by habitat in **a** Emboreet **b** Loiborsireet and **c** Oltukai village

A total of 1483 tsetse flies were caught in all surveyed villages, of which 1213 were *Glossina pallidipes*, 150 were *G. morsitans,* and 124 were *G. swynnertoni*. Generally, *G. pallidipes* was the most abundant species in riverine habitat in all surveyed villages while *G. swynnertoni* was the most abundant in the ecotone habitat (Fig. 3, Table 1). However, *G. morsitans* was the second most abundant and significant in all habitats across villages (Fig. 3, Table 1). The variation of three tsetse abundance patterns among habitats may be influenced by the quality of vegetation NDVI and the abundance of hosts (Additional file 2 and Additional file 3).

**Table 1:** Linear mixed effect models between the tsetse abundance (log x + 1) by species, as dependent variables, and habitats, as independent variables; the site is included as a random factor. a) Emboreet village b) Loiborsireet village and c) Oltukai village

	*G. pallidipes*		*G. swynnertoni*		*G. morsitans*	
Habitats	Coeff. ± SE	*P*-value	Coeff. ± SE	*P*-value	Coeff. ± SE	*P*-value
a)						
Intercept	−0.01 ± 0.03	0.7690	0.168 ± 0.06	0.0028	0.04 ± 0.05	0.4946
Ecotone	0.02 ± 0.03	0.6855	0.90 ± 0.07	0.0003	0.72 ± 0.06	0.0005
Open woodland	0.14 ± 0.04	0.6109	−0.09 ± 0.05	0.1925	0.01 ± 0.05	0.7561
Riverine	0.02 ± 0.02	0.0241	−0.14 ± 0.06	0.1102	−0.34 ± 0.06	0.5646
Wet season	0.02 + 0.02	0.4128	−0.04 ± 0.04	0.2512	0.006 ± 0.04	0.8642
Random effect SD: site	3.9E-06		2.4E-05		7.2E-0.6	
DF	261		261		261	
AIC	−128.84		148.77		109.76	
b)						
Intercept	0.46 ± 0.17	0.0154	0.1 ± 0.01	0.0001	0.14 ± 0.08	0.0604
Open woodland	−0.17 ± 0.23	0.5231	−0.04 ± 0.02	0.2001	−0.06 ± 0.09	0.5289
Riverine	1.79 ± 0.31	0.0103	0.02 ± 0.05	0.6371	−0.04 ± 0.12	0.7163
Wet season	0.61 + 0.1	< 0.0001	−0.07 ± 0.02	0.0019	0.11 ± 0.06	0.0562
Random effect SD: site	0.22		2.6E-06		0.07	
DF	209		209		209	
AIC	507.86		- 119.01		209.5	
c)						
Intercept	0.07 ± 0.19	0.7041				
Open woodland	0.16 ± 0.26	0.5756				
Riverine	0.85 ± 0.26	0.0477				
Wet season	−0.14 ± 0.06	0.0162				
Random effect SD: site	0.25					
DF	209					
AIC	276.5					

The abundance of tsetse species in other habitats is compared to swampy habitat

There was an inconsistent variation in tsetse species abundance pattern in open woodland and swampy habitats across villages (Fig. 3a and b) for Emboreet and Loiborsireet villages (Table 1a and b). However, only *G. pallidipes* species was found to infest Oltukai village habitats (Fig. 3c). There was incoherent relationship between tsetse species and seasons across villages. *G. pallidipes* had a significantly higher abundance in wet season for Loiborsireet and Oltukai villages (Table 1b, c). Nevertheless, *G. swynnertoni* only significantly had lower abundance in the wet season than dry season in Loiborsireet (Table 1b) while *G. morsitans* showed no significant variation between seasons across all villages.

There was significant variation in tsetse species among villages (Table 2). *G. pallidipes* was more abundant in Loiborsireet and Oltukai villages whilst *G. swynnertoni* dominated in Emboreet village (Table 2). Surprisingly, the two closely located villages, Emboreet and Loiborsireet were dominated by different tsetse species: *G. swynnertoni* and *G. pallidipes,* respectively (Table 2).

**Table 2:** Linear mixed effect models between the tsetse abundance (log x + 1) by species, as dependent variables, and village, as independent variables; the site is included as a random factor

	*G. pallidipes*		*G. swynnertoni*		*G. morsitans*	
Village	Coeff. ± SE	*P*-value	Coeff. ± SE	*P*-value	Coeff. ± SE	*P*-value
Intercept	0.061 ± 0.103	0.5515	0.176 ± 0.051	6E-04	0.12 ± 0.04	0.0027
Loiborsireet	0.991 ± 0.064	<0.0001	−0.105 ± 0.026	1E-04	0.059 ± 0.031	0.0561
Oltukai	0.187 ± 0.057	0.0010	−0.196 ± 0.023	<0.0001	−0.138 ± 0.027	<0.0001
Random effect: site	0.301		0.15			
DF	762		762		0.11	
AIC	1485		102.96		367.2	

Tsetse abundance of other villages is compared to Emboreet village

### Trypanosome infection rates in caught tsetse flies among habitats and villages

**Fig. 4:**
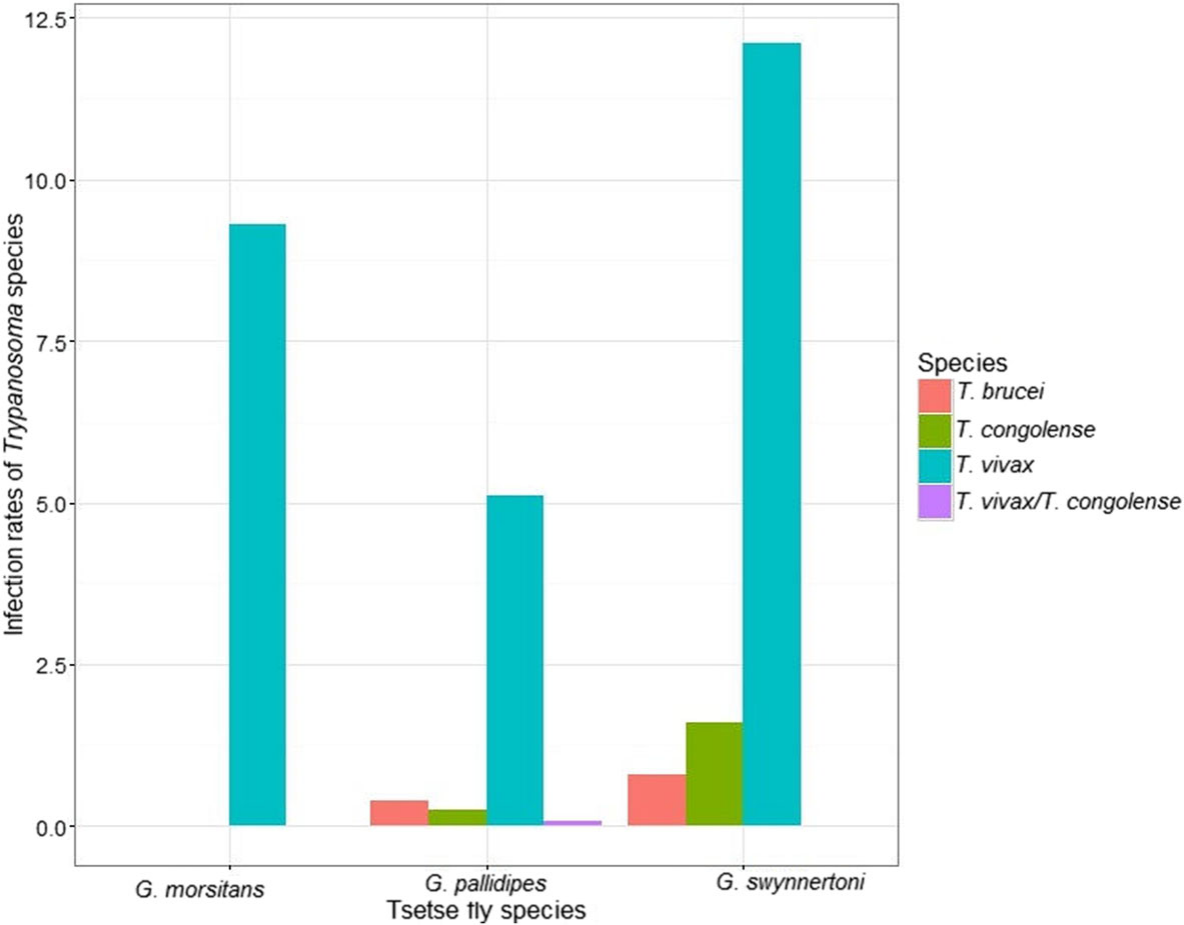
Infection rates of Trypanosoma species across various habitats of Maasai steppe

**Fig. 5:**
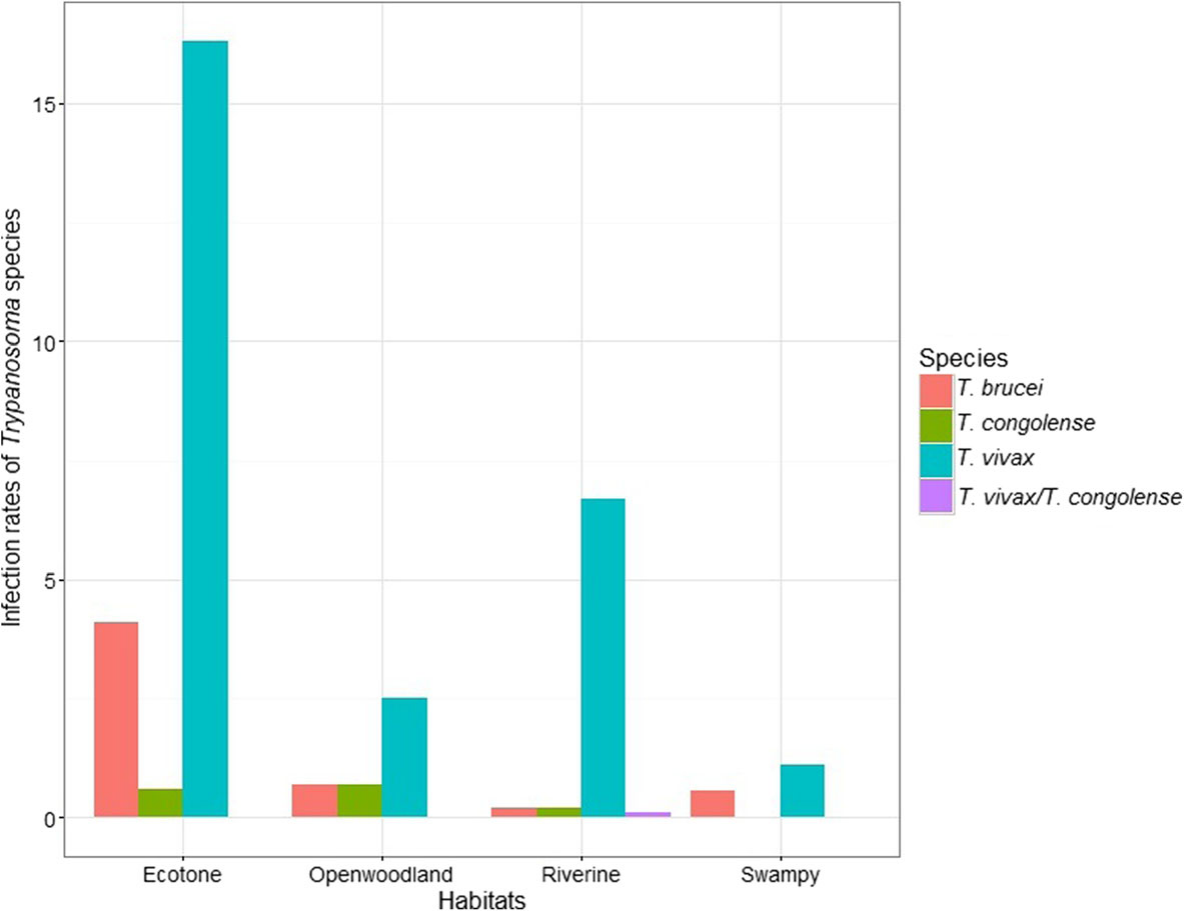
Infection rates of various Trypanosoma species detected among various species of tsetse flies

Out of 1483 flies collected 130 flies resulted positive to trypanosome infection with PCR analysis. The overall infection rate was 8.8%; the highest was found in *G. swynnertoni* species and the lowest was detected in *G. pallidipes* (Fig. 4). *Trypanosoma vivax* was the most common infection among the tsetse fly species (Fig. 4). The infection rates by trypanosome species showed spatial variation among habitats and villages. Specifically, ecotone 21.1%, open woodland 9.0%, riverine 6.8% and swampy habitat 7.7% and variation in the fly infection rates across villages: Emboreet 15.6%, Loiborsireet 5.5%, and Oltukai was 1.7%. Most of the fly infections were found during the dry season across all habitats, (χ^2^ = 0.421, DF = 3, *P* < 0.0001). *Trypanosoma vivax* was the most prevalent trypanosome species (92%) found in tsetse flies from all the habitats while *Trypanosoma congolense* (2.2%) and *Trypanosoma brucei* (4.4%) were less prevelent, few cases of co-infection (1.1%) were also observed in riverine habitat specifically from *G. pallidipes* (Figs. 4 and 5).

### Age structure of trapped tsetse flies

The estimated mean age of all tsetse caught was below 11 days. Based on the mean wing fray value of the age structure of caught flies during sampling, vector longevity in riverine habitat was the highest than flies caught from other habitats and mostly were *G.pallidipes* (Tables 3 and 4. The number and percentage of each Wingfrey category in relation to habitats and species are summarised in Tables 3 and 4. The overall mean wing fray value at which tsetse flies were detected with the infection was 1.6 which is equivalent to 11 days. However, there were differences in the wingfrey categories of infected flies among habitats and species (Tables 3 and 4).

**Table 3:** Age structure of trapped and infected tsetse species sampled during the dry and wet season collectively

Species	Wing fray categories, *n* (%)					Total	Age MWFV		Estimated age (days)
Trapped flies	1	2	3	4	5	6			
*G. pallidipes*	1060 (80)	71 (99)	35 (85)	25 (80)	13 (100)	6 (100)	1210	1.3	Under 11
*G. morsitans*	142 (11)	0 (0)	3 (7)	4 (12)	0 (0)	0 (0)	149	1.2	Under 11
*G. swynnertoni*	117 (9)	1 (1)	3 (7)	3 (8)	0 (0)	0 (0)	124	1.0	Under 11
Total	1319	72	41	32	13	6	1483		
Infected flies									
*G. pallidipes*	52 (61)	6 (100)	6 (100)	3 (100)	4 (100)	3 (100)	74	1.9	13 days
*G. morsitans*	14 (15)	0 (0)	0 (0)	0 (0)	0 (0)	0 (0)	14	1.0	Under 11
*G. swynnertoni*	19 (24)	0 (0)	0 (0)	0 (0)	0 (0)	0 (0)	19	1.0	Under 11
Total	85	6	6	3	4	3	107		

The numbers in brackets present percentage in the wingfrey category while the numbers outside the brackets present the number of tsetse flies in the wing free category. MWFV means mean wing fray value

**Table 4:** Age structure of trapped and infected tsetse sampled in different habitats during the dry and wet season

Habitats	Wing fray categories, *n* (%)						Total	Age MWFV	Estimated age (days)
Trapped flies	1	2	3	4	5	6			
Ecotone	140 (10)	3 (5)	1 (3)	2 (8)	1 (7)	0 (0)	147	1.1	Under 11
Riverine	139 (55)	64 (96)	30 (97)	24 (92)	14 (93)	7 (100)	878	1.4	Under 11
Open woodland	277 (21)	0 (0)	0 (0)	0 (0)	0 (0)	0 (0)	277	1.0	Under 11
Swampy	181 (14)	0 (0)	0 (0)	0 (0)	0 (0)	0 (0)	181	1.0	Under 11
Total	1337	67	31	26	15	7	1483		
Infected flies									
Ecotone	29 (34)	0 (0)	0 (0)	0 (0)	0 (0)	0 (0)	29	1.0	13 days
Riverine	43 (50)	6 (100)	5 (80)	3 (100)	4 (100)	3 (100)	64	2.0	Under 11
Open woodland	10 (12)	0 (0)	1 (20)	0 (0)	0 (0)	0 (0)	11	1.2	Under 11
Swampy	3 (4)	0 (0)	0 (0)	0 (0)	0 (0)	0 (0)	3	1.0	Under 11
Total	85	6	6	3	4	3	107		

The numbers in brackets present percentage in the wingfrey category while the numbers outside the brackets present the number of tsetse flies in the wing free category. MWFV means mean wing fray Value

Mean age of infected flies caught from riverine habitat was higher than others from the other habitats. Furthermore, our results show that only tsetse flies caught from ecotone had lower mean infective age compared to mean age of survival (Table 4).

We observed contrasting results for tsetse flies in riverine habitat, where mean wing fray value for infected flies was 2.0 equivalent to 14 days compared to mean longevity age of 1.4 which is below 11 days (Table 4). Similarly, findings were observed in age structure by species where mean age of *G. pallidipes* was higher compared to the same of the infected flies (Table 3). This suggests that many flies particularly *G. pallidipes*; the most abundant fly in riverine habitat dies without being infected by trypanosomes or reaching the infective stage.

## Discussion

The main objective of this study was to investigate the relative abundance and infection rates in various species of tsetse among habitats across villages of the Maasai steppe. In general, there were variations of tsetse species distribution patterns, abundance and infection rates in relation to habitat types and age.

On the question of influence of habitat on tsetse species abundance, we found a significant variation in habitat use by tsetse species across surveyed villages. *G. swynnertoni* which is endemic in northern Tanzania was observed to infest woodland-swampy ecotone characterized by frequent movement of wild animals, what suits mobile behaviour of this species of following moving objects [34]. On the other hand, the *G. pallidipes* infested riverine areas and higher vegetation areas of open woodland and swampy areas. These findings are in agreement with findings from other studies with similar savanna landscape in Africa [35–37]. However, two closely bordered villages were dominated by two different species of tsetse flies; Emboreet and Loiborsireet had higher proportions of two caught tsetse species; *G. swynnertoni* and *G. pallidipes,* respectively. It is probably because Loiborsireet had higher vegetation cover with many trees and tall grasses which provide suitable vegetation cover favouring pallidipes during wet season while Emboreet was dominated by open woodland and grassland. *G. pallidipes* catches in Oltukai village were caught in riverine areas.

The abundant patterns of different species of tsetse flies were significantly associated with NDVI except for *G. morsitans*. *G. swynnertoni* had a negative relation with NDVI indicating that there were low catches as vegetation cover increases which is in agreement with our previous work in press [38]. Nevertheless, *G. pallidipes* patterns increased with an increase in NDVI. This is probably because of high abundance of pallidipes in all habitats of Loiborsireet and Oltukai in riverine habitats where its abundance increased with increase of vegetation cover. This observation is in agreement with other studies which reported higher pallidipes abundance in the wet season with higher NDVI compared to dry season [39, 40]. Although, this study did not show a statistical significance between *G. morsitans* and NDVI, our findings may still have epidemiological importance.

We found that habitats with the highest number of host animals also had higher abundance of tsetse flies. Cattle play an important role in sustaining female tsetse to feed and pupate. This could be probably because of tsetse preference to feed on cattle [16, 41], increase in a number of cattle in the area and expansion in small-scale agriculture [21]. These factors consequently reduce both cattle grazing area which push grazing towards the shrinking wild-land and seasonal grazing patterns of wildlife [23]. In addition, other studies have shown changes of tsetse habitats through cultivation and other human activities leading to their elimination or tsetse appearing in what may be considered to be inappropriate habitats [20, 40]. This may be the case for Loiborsireet and Oltukai villages. However, the latter village has limited grazing area, thus farming and overgrazing have pushed livestock keepers to graze their animals in the permitted area of Manyara ranch, where we also documented most catches of tsetse. This is inconsistent with [40], that, land use changes in community land reduce habitat for host and tsetse and hence the conserved areas will remain to be the hotspot of trypanosome infection.

Trypanosome infection rates of various trypanosome species vary among habitats, village, tsetse species, and age. Ecotone and riverine habitats, which are dominated by *G. swynnertoni* and *G. pallidipes* in Emboreet and Loiborsireet villages, respectively were the most infected habitats in Maasai steppe. This is probably because of the abundant hosts in spite of seasonal wild animal migration inside the park especially during dry season, the time when livestock grazing patterns move towards the park boundary. This provides constant development for tsetse and circulation of trypanosomes in vector and vertebrate hosts. Lower trypanosome infection rates in swampy areas were limited by host availability especially during the wet season where animals move to the plains and during late dry season tsetse have limited shade to avoid desiccation. In spite of its highest abundance, *G. pallidipes* had lowest infection rates that could be contributed by variation in transmission of trypanosomes and vectorial capacity among tsetse species [34, 42, 43]. In addition, the infective age of flies based on mean wing fray value was higher compared to mean age of tsetse longevity in riverine habitats which were dominated by *G. pallidipes*.

*Trypanosoma vivax* was the most prevalent species in all infected tsetse flies compared to *T. congolense*, *T. brucei* and few observations of co-infections. This is probably because of biological differences of this species in development in tsetse flies. The midgut establishment of ingested trypomastigotes of *T. congolense* and *T. brucei* are notably limited by anti-trypanosomal factors (midgut trypanolysin and trypanoaglutinin) [44]. These factors fluctuate with normal digestive cycles and feeding patterns hence feeding at short interval stimulates their release and renders the fly refraction to infection. In addition, the longer periods of starvation renders the flies more susceptible to infection as agglutinin and lytic activity decrease with time. Furthermore, the migration of cyclic trypomastigotes from midgut loose ability to invade hypopharynx [45, 46]. Previous experiments showed up to 50–60% of *T. congolense* in morsitans persisted up to 30 days while they take 19–23 days to develop [47]. *Trypanosoma vivax* development takes place in the proboscis of the tsetse flies and persists up to 58 days [48]. *Trypanosoma vivax* also has unique differentiation in tsetse through loss of surface coat and 100% of its infection rate could be achieved with repeated feeding flies on an infected host [38].

The average age of the caught tsetse flies was below 11 days what provides a chance for *T. vivax* to circulate as its maturation takes 5 days at a temperature of 26 °C. This can also be used to explain the higher infections during dry season compared to wet season [49]. The longevity of the vector may be a limiting factor for the development of some trypanosomes requiring a longer time for maturation for example, *T. congolense* at 24 °C takes up to 15–20 days [34]. On the other hand, fly species differ in their infection rates and age at which tsetse become infective. In this study, there are clear differences in infection rates in *G. pallidipes* and *G. swynnertoni* which are mostly abundant in riverine and ecotone habitats respectively. This is probably because its mean infective age is higher than mean age of its survival and lower abundance levels of hosts, whereas, *G. swynnertoni* had low infective age to mean age of survival hence higher infection rates.

Although this study was limited to two seasons, the similarity of our findings with other studies suggests that observed patterns are significant to provide insight into the epidemiology of Trypanosomiasis in the area. The outcome of this study will provide insights in mapping hotspots of tsetse infestation and trypanosome infections. Since *T. vivax* is responsible for sylvatic transmission of trypanosomiasis, it opens avenues for further research on the role played by *Stomoxys* spp. and Tabanids in mechanical transmission of the infections.

## Conclusions

In summary, the study examined the spatial variation of abundance of tsetse flies and infection rates with trypanosomes among habitats, villages, tsetse species and age structure. The data suggests that the abundance of tsetse in various habitats was influenced by vegetation cover and host availability, while infection rates varied with the composition of tsetse species, species of trypanosome species, the age of fly to become infective in relation to the longevity of the tsetse species. The study lays groundwork for modelling tsetse spatial distribution patterns, the potential risk of trypanosomiasis transmission and plan for control of vector and disease.

## Additional files

Additional file 1: Multilingual abstracts in the six official working languages of the United Nations. (PDF 591 kb)

Additional file 2: Linear mixed effect models between the tsetse abundance (log x + 1) by species, as dependent variables, and NDVI as an independent variable; the site is included as a random factor. (CSV 306 bytes)

Additional file 3: Relationships between tsetse species abundance as a response variable and host species as explanatory variables. Only significant results are presented in this table. (CSV 465 bytes)

## Abbreviations

AIC: Akaike information criterion
Coeff.: Coefficient
DF: Degree of freedom
DNA: Deoxyribonucleic acid
EROS: Earth Resource Observation and Science Centre
IDRC: Canadian International Development Research Centre
ITS 1: Internal transcribed spacer 1
LME: Linear Mixed Effect model
MNP: Manyara National Park
MODIS: Moderate Resolution Imaging Spectrometer
MWFV: mean wing-fray value
NDVI: Normalized difference vegetation index
NM-AIST: The Nelson Mandela African Institution of Science and Technology
P: Probability significance value
PCR: Polymerase Chain Reaction
SE: Standard error
TDR: WHO Special Programme for Research and Training in Tropical Diseases
TNP: Tarangire National Pak
UNDP: United Nation Development Programme
UNICEF: United Nations International Children’s Emergency Fund
WHO: World Health Organisation
χ^2^: Chi-square
