# Supplementary material for: Patterns of tsetse abundance and trypanosome infection rates among habitats of surveyed villages in Maasai steppe of northern Tanzania
Source: Infect Dis Poverty. 2017 Sep 4;6:126. doi: 10.1186/s40249-017-0340-0 (PMC5582388; doi:10.1186/s40249-017-0340-0)
Supplement: Supplementary file 1 — Multilingual abstracts in the six official working languages of the United Nations. (PDF 591 kb) [file 40249_2017_340_MOESM1_ESM.pdf]

Translation of the abstract into the six official working languages of the United Nations

أنماط وفرة ذبابة تسي تسي ومعدلات عدوى المثقبيات بين موائل القرى التي شملتها الدراسة في سهول ماساي شمال تنزانيا

أنيباريكي نجونيوكا، بول س. غجاكيسا، أنا ب. إستيس، ليندا ب. سالكوا، السعادة ج. نكو، بيتر ج. هدسون، إيزابيلا م. كاتادوري

ملخص

خلفية: التغيرات في الغطاء الأرضي تسبب تغيرا في خصائص الموائل، والتفاعل العائل-الناقل وبالتالي معدلات الإصابة للعوامل المسببة للأمراض. في هذه الورقة، نورد التغيرات في أنماط توزيع ووفرة ومعدلات العدوى لذبابة تسي تسي فيما يتعلق بأنواع الموئل والعمر في سهول ماساي في شمال تنزانيا. وفي أفريقيا، أثر داء المثقبيات التي تنتقل عن طريق ذبابة تسيتسي سلبا على حياة الإنسان حيث يتعرض نحو 40 مليون شخص لخطر الإصابة بالمرض ما يسبب نتائج اجتماعية واقتصادية هائلة، مثل فقدان الماشية والإنتاجية الحيوانية والقوى العاملة.

أساليب: اصطدنا ذباب تسي تسي في مواسم جافة ورطبة بين أكتوبر 2014 ومايو 2015 في موائل مختارة عبر أربع قرى هي: إمبوريت ولويبورسيريت وكيموتوروك وأولتوكاي المجاورة للمناطق المحمية. وتشمل البيانات التي تم جمعها عدد وأنواع ذباب تسي تسي التي تم صيدها في الفخاخ الطاعنة، وتحديد تفاعل البوليميرز المتسلسل لأنواع المثقبيات واستخراج بيانات رصد مؤشر التغطية النباتية (NDVI) من مقياس الطيف التصويري المتوسط التحليل (موديس).

النتائج: تبين نتائجنا تباين وفرة أنواع ذبابة تسي تسي ومعدالت الإصابة بين الموائل في القرى التي شملتها الدراسة فيما يتعلق بمدى مؤشر التغطية النباتية ووفرة العائل. وقد أظهرت النتائج ارتفاع وفرة ذبابة تس تسي في مناطق تماس السنط والمستنقعات والموائل النهرية لإمبوريت وغيرها من القرى، على التوالي. وكانت وفرة تسي تسي غير متسقة بين الموائل في قرى مختلفة. كان إمبوريت شديدة الإصابة بـ (68٪ Glossina swynnertoni) في مناطق التماس والموائل المستنقعات تليها G. morsitans (28٪) G. pallidipes (4٪) في الموائل النهرية. وفي القرى المتبقية، كانت أنواع ذبابة تسي تسي السائدة بنسبة 95٪ G. pallidipes في جميع الموائل. Trypanosoma vivax كان أكثر الأنواع السائدة في جميع الذباب المصاب (95٪) مع عدد قليل من الملاحظات من الإصابات المشتركة (مع T. congolense أو T. brucei).

استنتاج: توفر نتائج هذه الدراسة إطارا لرسم خرائط النقاط الساخنة لعدوى ذبابة تسي تسي وداء المثقبيات، وتدعم المجتمعات المحلية للتخطيط للسيطرة الفعالة على داء المثقبيات.

Translated from English version into Arabic by Mahmoud Sami, through

TRANSLATORS
WITHOUT BORDERS

坦桑尼亚北部马赛草原受访村庄中采采蝇丰度和锥虫感染率的模式分析

AnibarikiNgonyoka, Paul S. Gwakisa, Anna B. Estes, Linda P. Salekwa, Happiness J. Nnko, Peter J. Hudson, Isabella M. Cattadori

摘要：

引言：土地植被的改变可以使栖息地的特征和宿主-媒介相互作用，以及由此引发的疾病感染率发生变化。本研究报告了坦桑尼亚北部马赛草原上采采蝇的分布模式、丰度和感染率的变化与栖息地的类型和采采蝇虫龄息息相关。在非洲，采采蝇传播的锥虫病对人类生活产生不利影响，

约 4 千万人口处于疾病的阴云中，并面临严重社会经济后果，例如牲畜、动物生产力损失和劳动力丧失等。
方法：在 2014 年 10 月-2015 年 5 月期间，我们在干、湿两季中分别从毗邻保护区的 4 个村庄（Emboreet、Loiborsireet、Kimotorok 和 Oltukai）捕获采采蝇。收集数据包括采采蝇的数量和种属信息，对其携带的锥虫进行 PCR 鉴定，并提取采用中等分辨率成像光谱仪（MODIS）监测的归一化植被指数（NDVI）数据。
结果：本研究结果表明，受访村庄中采采蝇物种丰度和感染率的差异与当地 NDVI 和宿主丰度相关。结果显示，Emboreet 村的 Acacia 沼泽群落交错区的采采蝇丰度较高，而其他村庄的河流区域采采蝇丰度较高。在不同村庄栖息地之间的采采蝇的丰度并不一致。Emboreet 村的群落交错区和沼泽地区丝舌蝇（*Glossina swynnertoni*）最多（68%），其次是河边的刺舌蝇（*G. morsitans*, 28%）和淡足舌蝇（*G. pallidipes*, 4%）。余下村庄中，淡足舌蝇是采采蝇中的优势族群，占 95%。最常见感染蝇类的是活动锥虫（*Trypanosoma vivax*, 95%），很少存在共感染现象，即与刚果锥虫（*T. congolense*）或布氏锥虫（*T. brucei*）共感染。
结论：本研究发现为制定采采蝇侵袭和锥虫感染热点提供了框架，有助于加强社区对锥虫病的有效控制。

Translated from English version into Chinese by Peng Song, edited by Pin Yang

CHINESE CENTER FOR DISEASE CONTROL AND PREVENTION
NATIONAL INSTITUTE OF PARASITIC DISEASES

Tendances de l’abondance tsé-tsé et des taux d’infection trypanosome dans tous les habitats des villages étudiés dans la Steppe Massaï du Nord de la Tanzanie

Anibariki Ngonyoka, Paul S. Gwakisa, Anna B. Estes, Linda P. Salekwa, bonheur J. Nnko, Peter J. Hudson, Isabella M. Cattadori

Résumé

Rappel des faits: le changements de la couverture terrestre modifie les caractéristiques de l’habitat, l'interaction hôte-vecteur et, par conséquent, les taux d’infection d’agents pathogènes. Dans cet article, nous rapportons les variations dans les modèles de distribution de tsé-tsé, l'abondance et les taux d'infection en en relation avec le type d’habitat et l’âge dans la Steppe Masaï du Nord de la Tanzanie. En Afrique, la trypanosomiase transmises par la mouche tsé-tsé a un impact négatif sur la vie humaine où environ 40 millions de personnes sont à risque de contracter la maladie, avec des conséquences socio-économiques dramatiques, par exemple, la perte de bétail, de la productivité animale et de la main d’oeuvre.
Méthodes: nous avons piégé les mouches tsé-tsé en saisons sèches et humides entre octobre 2014 et mai 2015 dans divers habitats de quatre villages: Emboreet, Loiborsireet, Kimotorok et Oltukai, adjacente aux zones protégées. Les données recueillies comprennent le nombre et les espèces de mouches tsé-tsé pris dans des pièges appâtés, l'identification de PCR des espèces de trypanosomes et l'extraction de données d’indice de végétation par différence normalisée (IVDN) par spectroradiomètre imageur à résolution moyenne (MODIS).

Résultats: nos résultats démontrent la variation du taux de l'abondance des espèces mouche tsé-tsé et du taux d'infection dans les habitats des villages étudiés en relation avec l'IVDN et l'abondance d'hôtes. Résultats ont montré une plus grande abondance de la mouche tsé-tsé en Acacia- écotone marécageux et habitats fluviaux pour Emboreet et d'autres villages, respectivement. L'abondance de la mouche tsé-tsé varie parmis les habitats dans différents villages. Emboreet a été fortement infesté de *Glossina swynnertoni* (68 %) dans l'écotone et habitats marécageux, suivie de *G. morsitans* (28 %) et *G. pallidipes* (4 %) dans des habitats lotiques. Dans les autres villages et dans tous les habitats, l'espèce dominante de mouche tsé-tsé, a été *G. pallidipes.* à 95 %. *Trypanosoma vivax* a été l'espèces la plus répandues dans toutes les mouches infectées (95 %) avec quelques observations de co-infections (avec *T. congolense* ou *T. brucei*).

Conclusion: Les résultats de cette étude offrent un cadre à la cartographie des zones à risque d'infestation tsé-tsé et d'infection de la trypanosomiase, et renforcer les communautés à planifier pour un contrôle efficace de la trypanosomiase.

Translated from English version into French by Pascale Salvatore, through

TRANSLATORS WITHOUT BORDERS

Закономерности распределения популяции мухи цеце и уровня заболеваемости трипаносомозом среди населения обследованных деревень в степи Масаи, на севере Танзании

Анибарики Нгоньока, Пол С. Гуакиса, Анна Б. Естес, Линда П. Салеква, Хэпинес Дж. Нко, Питер Дж. Хадсон, Изабелла М. Каттадори

Реферат

Справочная информация: Изменения почвенно-растительного покрова приводят к модификации как среды обитания, так и взаимосвязи между биологическими видами, являющимися хозяевами паразитирующих организмов и переносчиками заболеваний, а также, в конечном итоге, влияет на уровень инфецированности возбудителей заболевания. Данная работа представляет собой отчет об изменениях закономерности распределения и популяции мухи цеце, а также уровня инфецированности по типу и возрасту обитателей степи Масаи на севере Танзании. Трипаносомоз, переносчиком которого является муха цеце, оказывает негативное воздействие на человеческие жизни в Африке, где около 40 миллионов человек подвергаются риску заражения этой болезнью, влекущей серьёзные социально-экономические последствия, такие, как потеря скота, снижение продуктивности животных и утрата людских ресурсов.

Методы: В период с октября 2014 года по май 2015 года в избранных районах, охватывающих прилегающие к заповедным зонам четыре деревни: Эмборет, Лойборсирет, Кимоторок и Олтукай, были собраны образцы мухи цеце как во время засухи, так и в сезон дождей. Собранные данные включают в себя число и разновидности мухи цеце, собранные с

использованием ловушки с приманкой, идентификацию особей, являющихся переносчиками трипаносомоза произведенную с помощью ПЦР, извлечение данных наблюдения по Нормализованному относительному индексу растительности (NDVI) выполненное при помощи сканирующего спектрорадиометра среднего разрешения (MODIS).

Результаты: Полученные выводы свидетельствуют о вариации в популяции особей мухи цеце, а также об уровне инфецированности среди населения обследованных деревень по NDVI, а также по численности переносчиков заболевания. Результаты показали повышенную популяцию особей мухи цеце в болотистых экотонах Акации, а также в речном ареале обитания Эмборета и других деревень соответственно. Данные по численности мухи Цеце непоследовательны по средам обитания в разных деревнях. Эмборет оказался значительно инфецирован такими заболеваниями, как *Glossina swynnertoni* (68%) в экотоне и болотистых средах обитания, далее следует *G. morsitans* (28%) и*G. pallidipes* (4%) в речном ареале обитания. В остальных деревнях на 95% доминировала разновидность мухи цеце *G. Pallidipes* по всем средам обитания. *Trypanosoma vivax* оказалась наиболее распространённой разновидностью по всем зараженным мухам (95%) с рядом случаев сочетанной инфекции (с *T. congolense* или *T. brucei*).

Заключение: Выводы данного исследования обеспечивают основу для сопоставления очагов заражения мухи цеце и инфецированности трипаносомозом, а также способствует укреплению общин в разработке планов эффективного контроля за указанным заболеванием.

Translated from English version into Russian by Liudmila Tomanek (nee Volynets) , through

TRANSLATORS
WITHOUT BORDERS

Patrones de abundancia de la mosca tse-tse y tasas de infección por Trypanosoma en los hábitats de los poblados estudiados en la estepa masai del norte de Tanzania.

Anibariki Ngonyoka, Paul S. Gwakisa, Anna B. Estes, Linda P. Salekwa, Happiness J. Nnko, Peter J. Hudson, Isabella M. Cattadori

Resumen

Contexto: Los cambios en la cobertura vegetal modifican las características del hábitat, las interacciones huésped-vector y en consecuencia, las tasas de infección por agentes patógenos. En el presente artículo, se describen las variaciones de los patrones de distribución, la abundancia y las tasas de infección en relación con el tipo y edad de los hábitats, en la estepa masai del norte de Tanzania. En África, la tripanosomiasis, transmitida por la picadura de la mosca tse-tse, tiene un impacto negativo sobre la vida humana. Alrededor de cuarenta millones de personas se encuentran en riesgo de padecer esta enfermedad y las consecuencias socioeconómicas son dramáticas: pérdida de ganado, de productividad animal y de mano de obra.

Métodos: Entre octubre de 2014 y mayo de 2015, durante las estaciones seca y lluviosa, se capturaron moscas tse-tse en cuatro poblados: Emboreet, Loiborsireet, Kimotorok y Oltukai, todos ellos adyacentes a zonas de protección. Los datos recogidos incluyeron el número y la especie de las moscas tse-tse capturadas en trampas con cebo, la realización de una PCR para la identificación de la especie de Trypanosoma y la extracción de datos del NDVI (Índice Diferencial de Vegetación Normalizado) a partir del Espectrómetro de Formación de Imágenes de Resolución Moderada (MODIS).
Resultados: Nuestros hallazgos muestran la variación de la abundancia de las especies de moscas tse-tse y de las tasas de infección, en los hábitats de los poblados estudiados, y su relación con el NDVI y la abundancia de huéspedes. Los resultados mostraron una mayor abundancia de moscas tse-tse en el ecotono pantanoso de las Acacias, en Emboreet y en los hábitats ribereños, para el resto de poblados. La abundancia de moscas tse-tse fue inconsistente entre los hábitats de los diferentes poblados. El poblado de Emboreet resultó estar altamente infestado por *Glossina swynnertoni* (68%) en el ecotono y los hábitats pantanosos, seguida por *G. morsitans* (28%) y *G. pallidipes* (4%), en el hábitat ribereño. En el resto de poblados, la especie de mosca tse-tse dominante fue, en un 95% de los casos, *G. pallidipes,* en todos los tipos de hábitat. *Trypanosoma vivax* fue la especie más prevalente en todas las moscas infectadas (95%). Se observaron en algunos casos coinfecciones (con *T. congolense* o con *T. brucei*).
Conclusión: Los hallazgos de este estudio proporcionan un marco para el mapeo de zonas críticas de infestación por la mosca tse-tse y de infección por Tripanosoma y pueden resultar útiles para las comunidades que quieran llevar a cabo un plan de control eficaz de la tripanosomiasis.

Translated from English version into Spanish by Eila van Reck, through

TRANSLATORS
WITHOUT BORDERS
